# An experimental/numerical investigation of non-reacting turbulent flow in a piloted premixed Bunsen burner

**DOI:** 10.1007/s00348-021-03377-3

**Published:** 2022-01-24

**Authors:** Jhon Pareja, Timo Lipkowicz, Eray Inanc, Campbell D. Carter, Andreas Kempf, Isaac Boxx

**Affiliations:** 1 Institute of Combustion Technology, German Aerospace Center (DLR), 70569 Stuttgart, Germany; 2 Chair of Fluid Dynamics, Institute for Combustion and Gasdynamics (IVG), Universität Duisburg-Essen, Duisburg, Germany; 3 Air Force Research Laboratory, Wright-Patterson Air Force Base, Ohio, 45433 USA

## Abstract

**Abstract:**

In this paper, an experimental study of the non-reacting turbulent flow field characteristics of a piloted premixed Bunsen burner designed for operational at elevated pressure conditions is presented. The generated turbulent flow fields were experimentally investigated at atmospheric and elevated pressure by means of high-speed particle image velocimetry (PIV). The in-nozzle flow through the burner was computed using large-eddy simulation (LES), and the turbulent flow field predicted at the burner exit was compared against the experimental results. The findings show that the burner yields a reasonably homogeneous, nearly isotropic turbulence at the nozzle exit with highly reproducible boundary conditions that can be well predicted by numerical simulations. Similar levels of turbulence intensities and turbulent length scales were obtained at varied pressures and bulk velocities with turbulent Reynolds numbers up to 5300. This work demonstrates the burner’s potential for the study of premixed flames subject to intermediate and extreme turbulence at the elevated pressure conditions found in gas turbine combustors.

**Graphical abstract:**

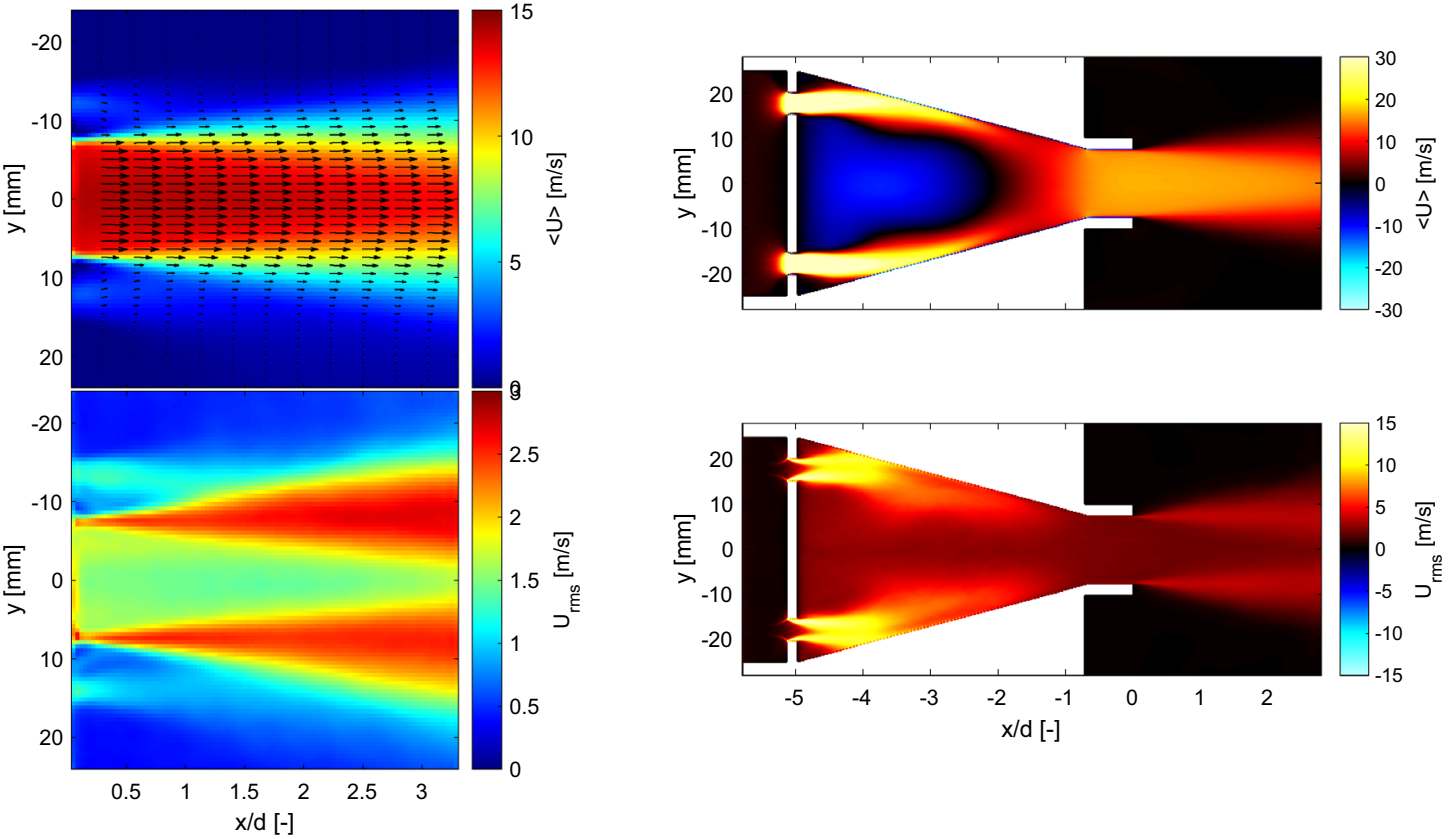

## Introduction

High-power combustion devices operate with highly turbulent flows of fuel and oxidizer to ensure proper mixing, and consequently, flames are subjected to extreme levels of turbulence. The effect of such turbulence on the structure and dynamics of the flames at practical conditions (i.e., high pressure and temperature) is not completely understood (Wabel et al. 2019). The study of premixed flames in combustion science has been essential for the development and improvement of suitable models to describe reaction processes in a given combustion system (Peters 2000). Moreover, lean premixed combustion has gained applicability in modern combustor concepts such lean, premixed pre-vaporized (LPP) gas turbines (GT) as a way to reduce pollutant emissions and improve thermal efficiency and scalability of the burners (McDonell 2016).

In premixed combustion, the level of turbulence is typically characterized by the turbulent Reynolds number based on integral scales ($Re_T$) and the Damköhler ($Da_{T,P}$) and Karlovitz ($Ka_{T,P}$) numbers defined by Peters (2000):

1 $$\begin{aligned}&Re_T = \frac{u'L_x}{\upsilon }, \end{aligned}$$

2 $$\begin{aligned}&Da_{T,P} =\frac{S_LL_x}{u'\delta _{L,P}}, \end{aligned}$$

3 $$\begin{aligned}&Ka_{T,P} = \left( \frac{u'}{S_L}\right) ^{3/2}\left( \frac{\delta _{L,P}}{L_x}\right) ^{1/2}, \end{aligned}$$

where $u'$ is commonly calculated as the root-mean-squared (rms) velocity fluctuations of the flow, $L_x$ is the longitudinal integral scale, $\upsilon$ is the kinematic viscosity of the reactants, $S_L$ is the unstretched laminar burning velocity and $\delta _{L,P}$ is the laminar flame thickness. Large-scale GT for power generation operate with enclosed flames at elevated pressures (pressure ratios up to 40 (Lachaux et al. 2005)) and turbulent flows with $u'$ values up to 50 m/s and $L_x \ge$ 1 cm (Gicquel et al. 2012). This results in flow conditions with large $Re_T$ ($\ge$ 100,000) and $Ka_{T,P}$ ($\sim O$ $\cdot$ 10$^2$ to 10$^3$), and low $Da_{T,P}$ ($\le$ 1). Modelling such flames and mimicking those conditions at laboratory scale to study turbulence/flame interactions is very challenging.

Turbulent premixed flames are frequently modelled based on the flamelet concept (Peters 2000) in which the turbulent flame front is assumed to be an infinitely thin layer containing both a preheat and a reaction region. The thermochemical state of a flamelet is similar to the one of a laminar flame (Peters 2000). Establishing the range of conditions over which flamelet-like models are valid is essential for the practical application of combustion simulations. According to combustion theory, flames at GT conditions are expected to operate in a regime in which the preheat region is broadened and the reaction region is thin (i.e., it is not expected to deviate from $\delta _{L,P}$) (Peters 2000; Gicquel et al. 2012; Skiba et al. 2018). To validate such prediction, experiments and direct numerical simulations (DNS) are necessary for detailed turbulent flame characterizations. Recently, Driscoll et al. (2020) provided a comprehensive review on experimental and DNS databases of premixed flames subjected to extreme turbulence. They classified the available databases as intermediate and extreme turbulence levels based on $Re_T$ and $Ka_{T,P}$. According to their definition, extreme turbulence is the condition for which $Re_T$ exceeds 2800 or $Ka_{T,P}$ is over 100 that results in broadened flamelets.

Regarding experiments, canonical premixed flames subjected at high levels of turbulence are studied using Bunsen burners such as those of Michigan Hi-Pilot (Temme et al. 2015) and Toronto (Tamadonfar and Gülder 2014), jet burners such as the premixed piloted jet burners of Sydney (PPJB) (Dunn et al. 2007; Smolke et al. 2017) and Lund (LUPJ) (Zhou et al. 2017), and the LBL low swirl burner (Cheng et al. 2009). These burners usually operate at atmospheric pressure. Therefore, high $Re_T$ values are achieved using turbulence generators and high bulk velocities. A more challenging (but also more technically relevant) alternative is to maintain bulk velocities and turbulence intensities similar to those found in a GT combustors and increase the chamber pressure to match the thermochemical properties of the flames to be modelled (Griebel et al. 2007; Venkateswaran et al. 2014).

There is still a lack of experimental data on turbulent premixed flames at elevated pressures. Available experiments include spherical flames (Jiang et al. 2016) and Bunsen-type burners enclosed in high-pressure combustion chambers (Lachaux et al. 2005; Ichikawa et al. 2011; Fragner et al. 2015). However, most of the studied conditions cover low and intermediate turbulence levels. Few experimental characterizations of turbulent flames have been performed at elevated pressures and at conditions with high values of $Re_T$ or $Ka_{T,P}$ (Cheng et al. 2009; Venkateswaran et al. 2014).

In this context, the present work introduces a new axisymmetric piloted Bunsen burner designed for the study of premixed flames subjected to intermediate and extreme turbulence levels at high pressure. The aim of this study is to characterize the (non-reacting) turbulent flow generated by the burner at atmospheric and elevated pressure conditions using high-speed particle image velocity (PIV). The experimental results are compared to a large eddy simulation (LES), both to demonstrate the robust predictability of the burner turbulence characteristics, and also to study the in-nozzle flow phenomena responsible for achieving it.

## Materials and methods

### The DLR Bunsen burner

**Fig. 1:**
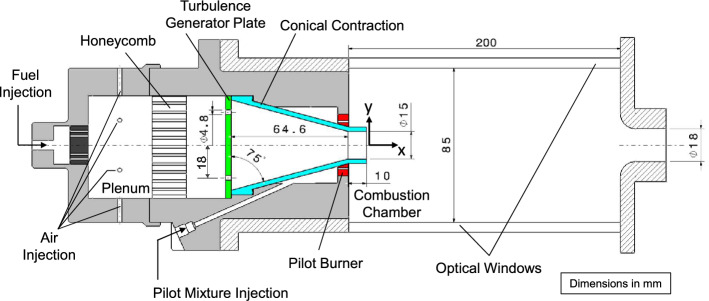
Schematic cross section of the DLR Bunsen burner and combustion chamber

Figure 1 shows a schematic cross section of the piloted premixed Bunsen burner. Reactants (air and fuel) are injected and mixed in a cylindrical plenum (diameter 50 mm $\times$ length 31.5 mm). Air is injected to the mixing section radially through 6 holes (5 mm in diameter). Fuel is injected axially to the mixing section through an array of 12 holes of 1 mm in diameter. After the mixing section, reactants pass a honeycomb flow-straightener to the turbulence generation assembly which consists of a high-blockage ratio plate and a conical contraction. The internal geometry is similar to the plate-nozzle system of Coppola and Gomez (2009). The conical contraction has a contraction angle of 15$^\circ$, a straight section of 10 mm and an outlet diameter of 15 mm. The turbulent generator plate located at the base of the conical contraction has 3 mm in thickness with four circular holes (4.8 mm in diameter) evenly spaced drilled in a ring of 36 mm in diameter which results in a blockage ratio of 96$\%$. The high-blockage ratio was designed to yield geometrically simple boundary conditions for numerical simulations.

For both the atmospheric and the elevated pressure experiments, the burner was enclosed in a combustion chamber with a cross section of 85 $\times$ 85 mm, a length of 200 mm and contoured exit nozzle of 18 mm in diameter. The chamber provides optical access from four sides. For measurements at atmospheric pressure, the burner exhausts into the laboratory; for measurements at elevated pressure, same device is mounted in the DLR high-pressure optical test-rig (HIPOT) for gas turbine model combustors (Boxx et al. 2015; Slabaugh et al. 2016). The HIPOT consists of a pressure vessel capable of operating at up to 30 bars, with thermal loads up to 300 kW. The test rig supplies up to 200 g/s of combustion air, with preheating option up 673 K. The entire test rig is mounted on a three-axis translation stage.

### Experimental flow conditions

The experimental study of the turbulent flow at the burner exit was carried out for non-reacting flow. Therefore, only air flowed through the burner. Table 1 summarizes different operational conditions used to study the turbulent flow fields generated with the burner. At atmospheric pressure and room temperature, an air mass flow of 2.85 g/s resulted in a bulk exit velocity $U_0$ = 13.4 m/s. At high pressure, the turbulence characteristics of the flow were evaluated at constant pressure of *P* = 5 bar and temperature *T* = 293.15 K while the air mass flow was adjusted to sweep the bulk velocity at the nozzle exit ranging from 7 to 20 m/s. The effect of pressure on the turbulent field properties was evaluated at a constant (bulk flow) exit velocity of 20 m/s by varying the combustion chamber pressure from 3 to 9 bar.

**Table 1:** Air mass flow $\dot{m}_{air}$ [g/s] used at different bulk velocities and pressure conditions. For all conditions *T* = 293.15 K

*P* [bar]	$U_0$ [m/s]				
	7	10	13.4	15	20
1	-	-	2.85	-	-
3	-	-	-	-	12.86
5	7.45	10.65	-	15.97	21.29
7	-	-	-	-	29.81
9	-	-	-	-	38.32

### High-speed PIV measurements

Instantaneous velocity fields were measured at several locations downstream of the nozzle exit via particle image velocimetry (PIV). Measurements were performed at atmospheric and elevated pressured. Titanium dioxide ($\hbox {TiO}_2$) particles ($\sim$1 $\mu$m in size) were seeded to the flow using the air supply system. For the atmospheric pressure experiments, the tracer particles were illuminated using a dual-cavity, diode-pumped frequency-doubled solid state laser system (Edgewave IS 6IIDE, $\sim$ 2.6 mJ/pulse, 7.5ns pulse duration). The laser beam was expanded using a cylindrical telescope to a thin sheet of 48 mm height at the measurement volume. Mie scattering signals from the seeding particles were collected perpendicularly to the laser sheet using a high-speed CMOS camera (LaVision HSS6) coupled with a 100-mm objective lens (f/5.6, Tokina AT-X Macro). The camera was operated in dual-frame mode with an array size of 768 $\times$ 768 pixel and a field of view (FOV) of 50 $\times$ 50 mm. Images were acquired at a frame rate of 5 kHz using a $\Delta$ $t =$ 20 $\mu$s. Onboard memory of the camera system enabled the acquisition of 9700 images (1.94 s) per run.

For the elevated pressure experiments, illumination was performed with a dual-cavity, diode-pumped solid-state frequency-doubled laser system (Edgewave, IS200-2-LD) at 10 kHz. The laser sheet had a height of 25 mm at the measurement volume. The inter-pulse time was set in accordance with each bulk velocity condition. The signal was collected with a high-speed CMOS camera (LaVision HSS8), equipped with a 200-mm objective lens (f/5.6Nikon, AF-Micro Nikkor). The camera array size was 704 $\times$ 520 pixel, and the corresponding FOV was 22 $\times$ 16.4 mm. A total of 30,000 image pairs were recorded at 10 kHz for an acquisition time of 3 s.

Velocity vectors were computed via cross-correlation of image pairs with a multi-pass adaptive window offset cross-correlation function using a commercially available software package (LaVision Davis 10). The final interrogation window size was 16 $\times$ 16 pixels with an overlap of 50%. The resulting spatial resolution of the velocity measurement was 0.52 mm/vector and 0.25 mm/vector for the atmospheric and elevated pressure measurements, respectively. The uncertainty of the single-shot PIV measurement was calculated using Davis 10 as $\sim$3%. The statistical uncertainty of the mean velocity fields lied between 0.2 and 0.5% of the average value.

### Simulations

The large eddy simulations (LES) were performed with the in-house code *PsiPhi* (Kempf et al. 2011) to solve the Favre-filtered governing equations for mass and momentum in a Low-Mach number approach. The effect of the sub-filter velocity fluctuations was modelled with an eddy-viscosity assumption, while the sigma model by Nicoud (2011) was used to compute the turbulent viscosity. *PsiPhi* uses a distributed memory approach for communication with aid of the message passing interface (MPI) in a non-blocking formulation to perform computations and communication simultaneously. Continuity was ensured by a fractional-step projection method, where the Poisson equation was solved with a conjugated complex solution algorithm. The equations were discretized in terms of finite volumes on an equidistant Cartesian grid with a total number of 67 million numerical cells at a grid resolution of 0.2 mm. Convective fluxes were interpolated with central difference schemes for momentum and a total variation diminishing (TVD) scheme for scalars employing the CHARM limiter. An efficient immersed boundary technique was applied to consider the inner burner geometry.

The simulation domain included the inner geometry of the turbulent generator plate and conical contraction. The size of the domain in the axial direction was 128.6 mm, starting 10 mm upstream of the turbulent generator and extending to 42 mm downstream of the nozzle exit. The transversal cross section of the domain had a size of 64.6 $\times$ 64.6 mm. The velocities at the inlet were fixed to match a velocity at the nozzle exit of 15 m/s. A Dirichlet boundary condition for pressure and Van Neumann (zero gradient) for all other quantities were used. In contrast to compressible solvers, there is no risk of pressure wave reflections. For simplicity and to reduce the computational costs, the domain downstream of the nozzle exit was not enclosed and the pilot flow was not included in the simulations.

## Results and discussion

### Turbulent flow field at atmospheric pressure

**Fig. 2:**
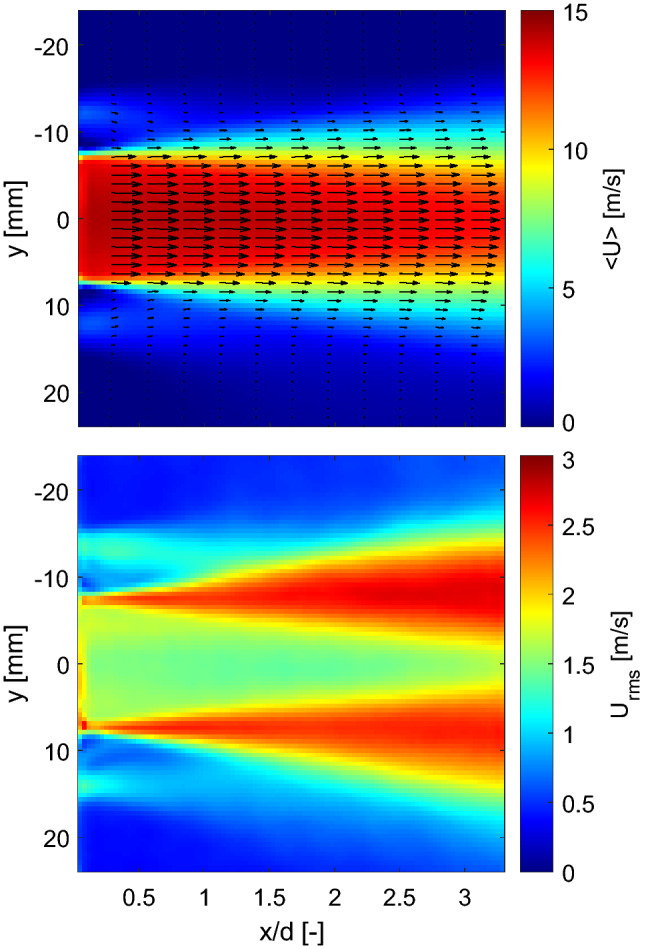
Resulting mean (top) and rms (bottom) fields of the axial velocity component measured at atmospheric pressure, at the middle longitudinal plane of the burner. For visualization, every 2^nd^ and 8^th^ vector is plotted along the radial and axial directions, respectively

Figure 2 presents the experimental results of the mean, $<\!U\!>$, and rms, $U_{rms}$, fields of the axial velocity component measured at atmospheric pressure, downstream of the nozzle exit at the middle longitudinal plane of the burner. All results are spatially referred to a *xy*-coordinate system located at the nozzle exit of the burner (see Fig. 1). These PIV results reveal a high symmetry of the axial velocity and its fluctuations. The turbulence intensity is defined here, based only the axial component, as $U_{rms}/U_0$. For this particular operating condition, the turbulence intensity is approximately 11% at locations of interest for premixed flames (i.e., around the jet center).

**Fig. 3:**
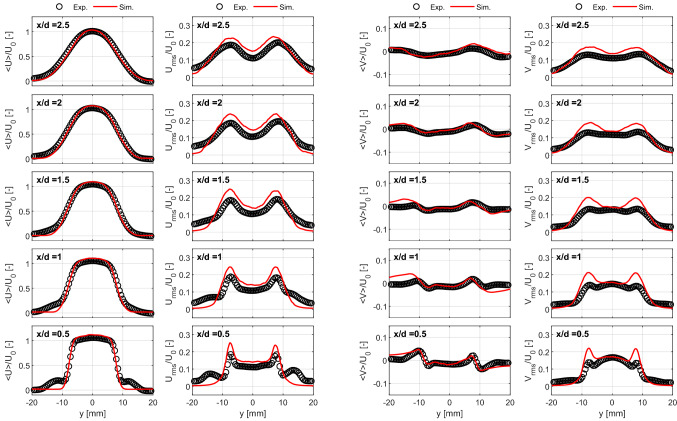
Mean and rms radial profiles of the axial (left) and radial (right) components of the velocity at different downstream locations from PIV measurements (symbols) and LES simulations (solid lines). Magnitudes normalized by the corresponding $U_0$ values

To evaluate the predictability of the burner in terms of the turbulent flow field characteristics, the experimental results at the nozzle exit were compared with those from LES. For this purpose, Fig. 3 shows radial profiles of the mean and fluctuations of the axial and radial components of the velocity at different locations downstream of the burner. Because experiments and simulations were performed independently at slightly different bulk velocities, the magnitudes are normalized by the corresponding $U_0$ values. The comparison of the magnitude of the mean axial and radial velocity components shows that the axial component dominates the flow. The mean axial velocity at the burner exit (*x*/*d* = 0.5) is reasonably uniform across most of the exit flow. The fluctuations of the axial velocity are reasonably constant at the center of the jet. In contrast, the profile of radial velocity component around that same location has a slightly curved shape.

As can be observed, in general, LES captures the magnitude and shape of the mean and fluctuating components of the axial and radial velocities of the turbulent flow generated by the burner. Because the pilot flow was not included in the LES, small differences between the measured and predicted values can be observed near the burner exit at radial locations around *y* = ±10 mm. Additionally, the peaks of the fluctuations at the shear layer are slightly overpredicted by the simulations. This could be related to the open jet configuration used for the simulations contrarily to the closed one of the experiments.

**Fig. 4:**
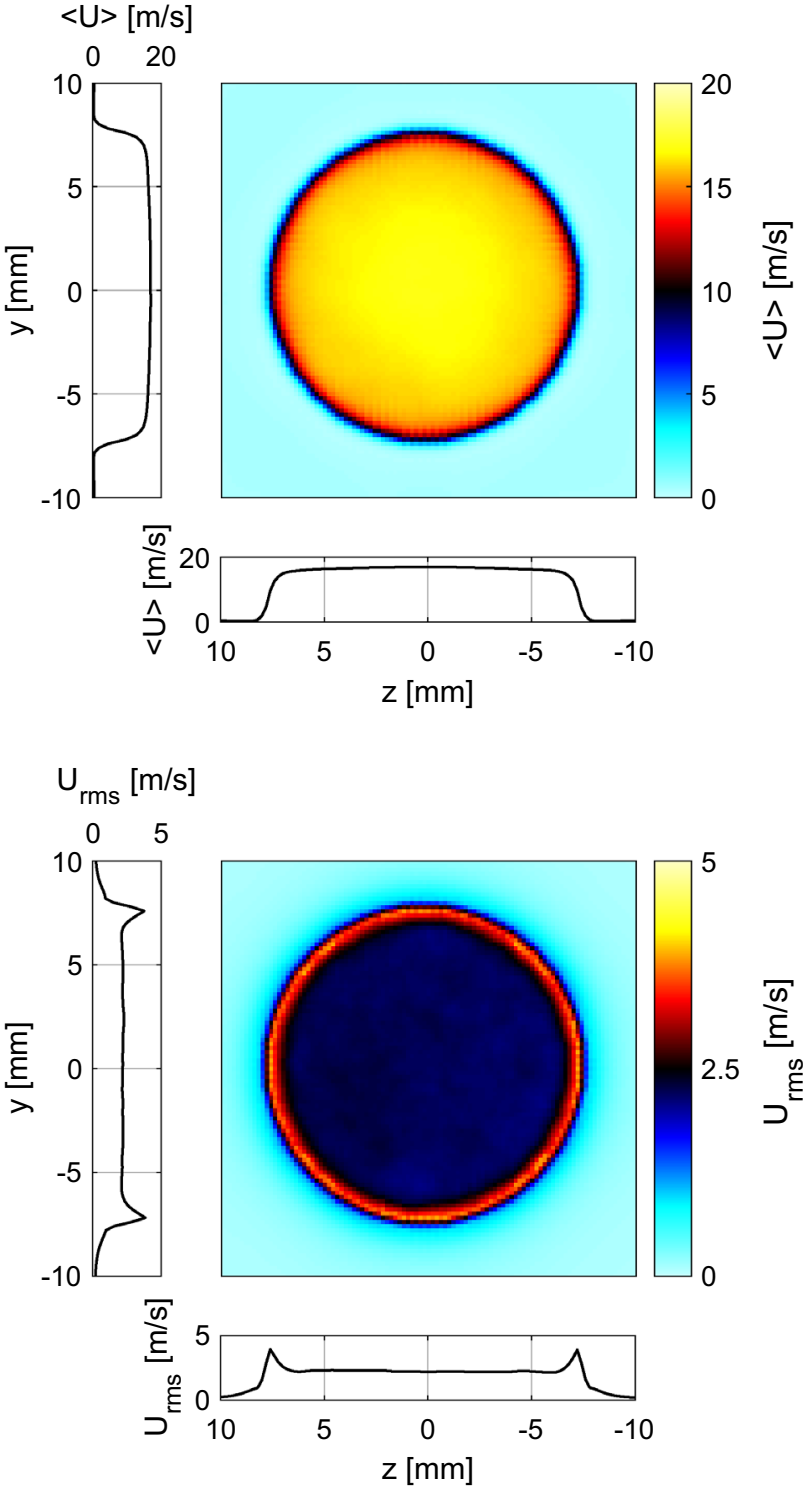
Mean (top) and rms (bottom) fields of the axial velocity component at the traverse plane at the burner exit. Vertical and horizontal profiles at center of the burner exit are included

Figure 3 indicates the LES computation accurately reproduces the magnitude and shape of the mean and fluctuating profiles of the axial and radial velocity with increasing downstream distance from the nozzle exit. It is therefore reasonable to assume that it is accurately replicating the 3D mean and fluctuating flow at the nozzle exit. Figure 4 shows mean (top) and rms (bottom) fields of the axial velocity component at the *yz*-plane, directly at the burner exit. Consistent with the profiles shown in Fig. 3, the flow exhibits reasonable homogeneity. Furthermore, the profiles of mean and fluctuating velocity plotted in Fig. 4 show a high degree of radial and azimuthal symmetry.

**Fig. 5:**
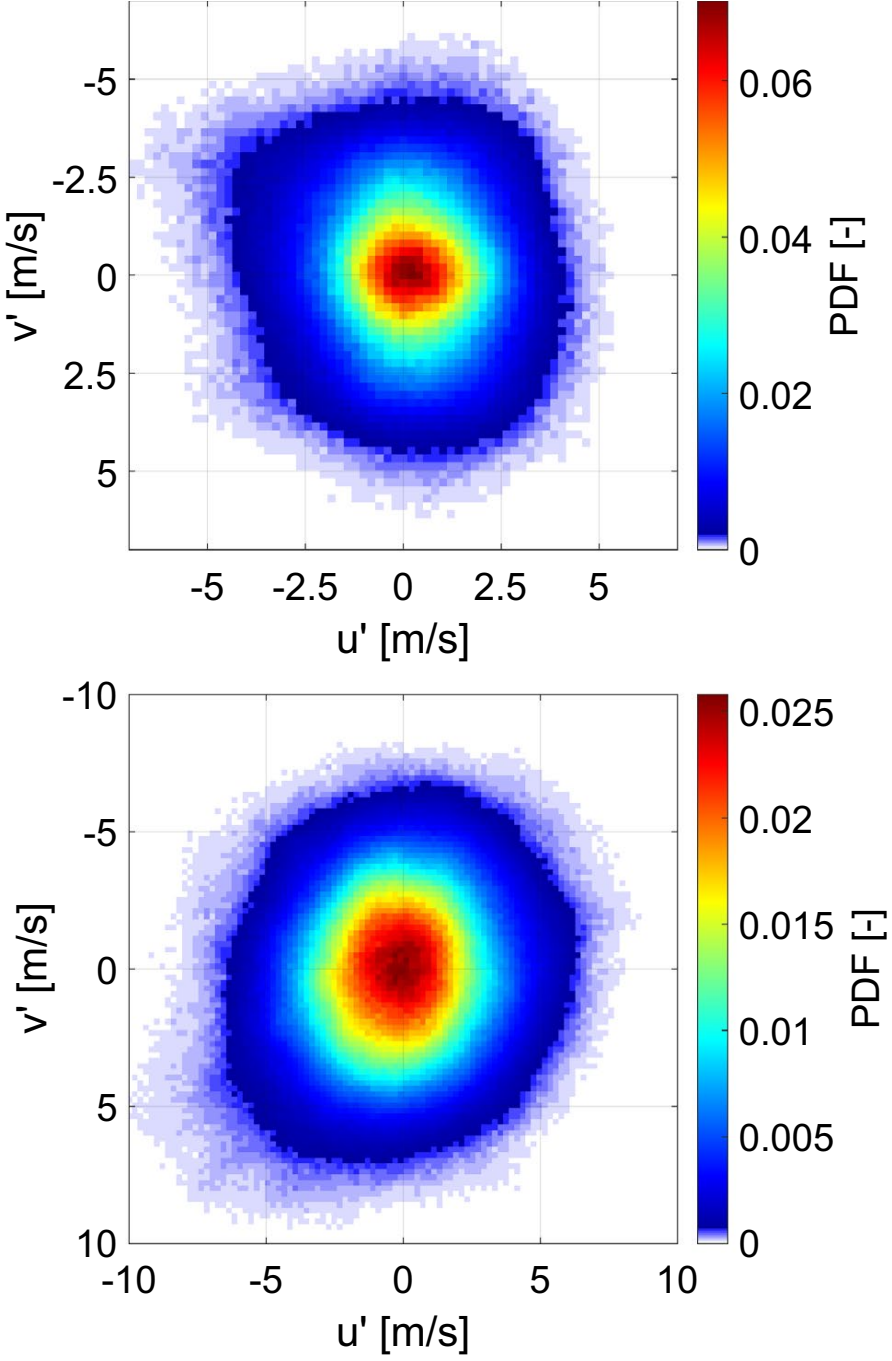
Joint probability density function (PDF) of axial and radial velocity fluctuations from experimental (top) and simulation (bottom) results

The isotropy of the turbulence generated at the burner exit was statistically evaluated in terms of the local fluctuating velocities for a region of the jet core excluding the outer shear layer (10 mm $\times$ 20 mm along the radial and axial directions, respectively). The local axial ($u'$) and radial ($v'$) velocity fluctuations were compared using the joint probability density function (PDF) as displayed in Fig. 5 for both experimental and simulation results. The PDFs were computed using 1000 statistically independent single shots. For each location of each single-shot, the local fluctuations were calculated as the local deviation from the mean velocity ($u' = U-<\!U\!>$ and $v' = V-<\!V\!>$). The joint probabilities for both PIV and LES results, represented by the intensity maps of Fig. 5, are centered around $(u',v')$ = 0 and do not exhibit a preferred orientation. This indicates a reasonable degree of isotropy of the turbulent flow. The moderately larger fluctuations of the simulation results are in agreement with the differences observed in Fig. 3.

**Fig. 6:**
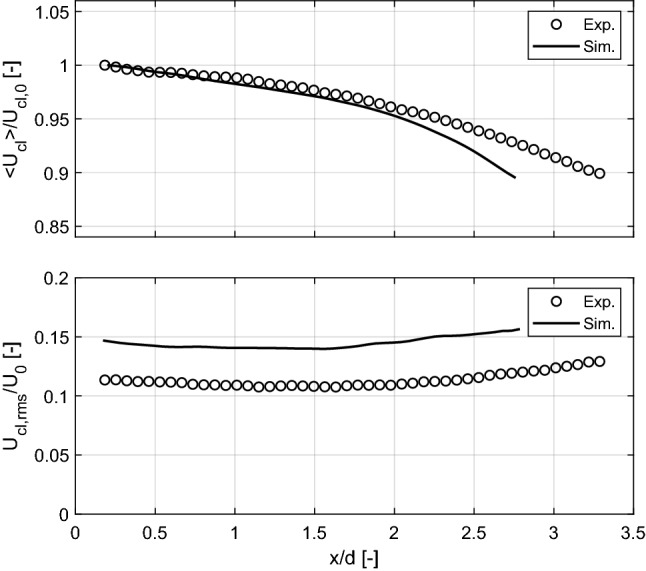
Normalized axial profiles of the mean (top) and fluctuating (bottom) velocities along the centerline of the flow from experimental (symbols) and simulations (solid lines) results

A more detailed examination of the behavior of the turbulent flow in the axial direction is performed by analyzing the normalized profiles of the mean and fluctuating velocities along the centerline of the flow. Figure 6 (top) compares the mean centerline axial velocity, for both experiments and simulations, normalized by the corresponding centerline velocity at the nozzle exit $U_{cl,0}$. It can be seen that the LES computation accurately replicates the measured values out to approximately $x/d \approx$ 2. Regarding the velocity fluctuations, $U_{rms}$ is normalized by the corresponding exit bulk velocities $U_{0}$ in Fig. 6 (bottom). In this case, $U_{rms}/U_0$ slowly decays near the burner exit down to $x/d \approx$ 1.5. After this location, the normalized $U_{rms}$ starts increasing again. This behavior is well predicted by the LES results as well. Similar tendencies for a decay/recovery of the turbulent fluctuating velocity have been previously reported for turbulent non-reacting jets (Kim et al. 2020; Fragner et al. 2015). According to Fig. 6 (bottom), the turbulence intensity at the centerline of the flow was fairly constant at $\sim$11.5% for the experiments and $\sim$14% for the operating conditions of the simulations, at locations down to *x*/*d* = 2.

The profiles Fig. 6 (top) demonstrate that the LES computation accurately reproduces the mean centerline velocity decay for the region 0 $< x/d<$ 2, but overestimates the magnitude of the velocity fluctuations by approximately 20% for the same locations. It is interesting to note, however, that the shape of the centerline profile of velocity fluctuations is quite well replicated by the LES. Although we were unable to establish conclusively the cause of these differences, a closer inspection of the profiles of mean and fluctuating velocity in Fig. 3 suggests a plausible explanation.

The PIV measurements were conducted with the burner mounted in an optically accessible confinement chamber. In addition, a stream of non-reacting room-temperature air was passed through the pilot ring of the burner. The use of a confinement chamber was motivated by the design goal of eventually operating the burner in a high-pressure combustion test-rig, wherein confinement of the flame is essential. The goal of the non-reacting pilot air was to isolate the flow at the nozzle exit from the possible oscillations originating within the recirculation zones that form at the corners of the optical confinement chamber. As the goal of the present study was to characterize and understand the in-nozzle turbulent flow characteristics of the burner, the (substantial) additional computational cost of fully replicating these two flow features was not considered justifiable.

The profiles of mean and fluctuating velocity shown in Fig. 3 show that the LES computation accurately predicts the values measured at the centerline for *x*/*d* = 0.5, but significantly underpredicts both at the jet periphery. This is clearly indicative of the lack of a pilot flow in the simulation. The differences in measured and computed profiles of mean velocity at the jet periphery diminish rapidly with downstream distance. The differences in fluctuating velocity at the jet periphery, however, increase with downstream distance. This is consistent with the (expected) effect of a low velocity co-flow acting to stabilize the shear-layer of the jet. The under-prediction of mean centerline velocity decay at $x/d>$ 2 is consistent with the combustion chamber acting to restrict somewhat the free expansion of the jet. As confinement effects scale with the jet-to-confinement area ratio, one would certainly expect (consistent with our observation from Fig. 6) the effect to become more pronounced with increasing downstream distance from the nozzle exit.

Taken together, the differences in profiles of measured and computed velocity at the burner centerline in Fig. 6 demonstrate the importance of careful consideration of boundary conditions in burners designed for testing and validation of numerical models. Future studies of the burner described in this work should certainly account for features such as confinement and pilot flow around the nozzle exit.

The spatial and temporal information provided by the PIV measurements and LES allow the direct calculation of turbulent length scales using the spatial autocorrelation function. For the present work, a normalized, two-dimensional spatial autocorrelation function for the axial velocity component was computed at the location ($x_0$,$y_0$) as:

4 $$\begin{aligned}&r(x,y)=\frac{\frac{1}{N}\sum _{1}^{N}u'(x,y) \times u'(x_0,y_0) }{U_{rms}(x,y) \times U_{rms}(x_0,y_0)}, \end{aligned}$$

where $u' = U-<\!U\!>$ and *N* is the total number of 2D single shots used for the computation.

**Fig. 7:**
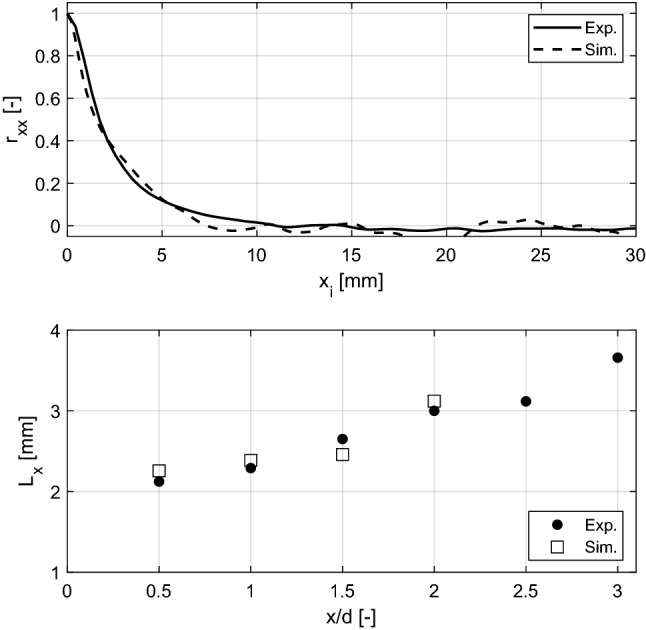
Top: normalized autocorrelation function of the axial velocity component along the axial direction at *y* = 0 and *x*/*d* = 0.5. Bottom: variation of the longitudinal integral scale with the distance from the burner exit

Figure 7 (top) shows the normalized autocorrelation function of the axial velocity along the axial direction ($r_{xx}$), calculated for a point on the centerline of the velocity field ($y_0$ = 0) at $x_0/d$ = 0.5 using data from both measurements and simulations. In this case, the horizontal axis is the relative distance to point of interest ($x_i = x-x_0$). As it can be seen, both curves decay to zero within the corresponding domains. Therefore, the longitudinal integral scale $L_x$ can be calculated by integrating the area under each curve. The resulting $L_x$ was 2.12 mm and 2.26 mm for the experimental and LES data, respectively. An additional check was performed taking advantage of the high temporal resolution of the LES results, and the integral time scale was calculated using the temporal auto-correlation and Taylor’s frozen flow hypothesis as 2.24 mm. With these results and using Eq. (1), turbulent Reynolds numbers, $Re_T$ = 214 and 316 were calculated for the experimental and simulated turbulent flows, respectively. These values are in good agreement with previously reported turbulent flows at atmospheric pressure using similar turbulence generation mechanisms at comparable bulk velocities (Tamadonfar and Gülder 2014; Kim et al. 2020).

The procedure for calculating $r_{xx}$ and $L_x$ was repeated for points located on the centerline of the velocity field at different locations downstream of the nozzle exit. The results are summarized in Fig. 7 (bottom) where a very good agreement between the scales calculated with the experimental and LES data is evident. The longitudinal integral length scale grows with the increase in the distance from nozzle exit, in agreement with previously reported data for non-reacting turbulent flows (Coppola and Gomez 2009; Khashehchi et al. 2013; Kim et al. 2020). In summary, a nearly isotropic turbulent flow is generated inside the Bunsen burner and the turbulent scales are transported to locations where reactions are expected to occur (Carbone et al. 2017; Cheng et al. 2009).

**Fig. 8:**
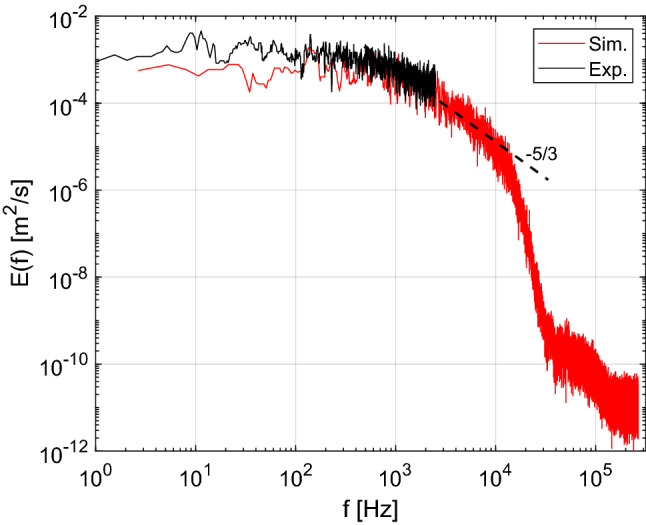
Normalized turbulent kinetic energy spectra from 5 kHz PIV measurements and LES data at *x*/*d* = 0.5

An additional evaluation of the turbulent flow characteristics was performed by computing spectrum of the turbulent kinetic energy. The spectra shown in Fig. 8 were calculated from both experimental and simulation data near the burner exit (centerline, x/d = 0.5). The measured spectrum is limited by the 5 kHz sampling rate of the PIV measurements. The profile calculated from the LES results follows the expected -5/3 slope of well-developed turbulence over at least a decade in frequency space. The lack of any strong local spikes indicates that there were no coherent oscillations in the flow at the nozzle exit (such those from precession or jet flapping).

### LES results: inner flow observations

**Fig. 9:**
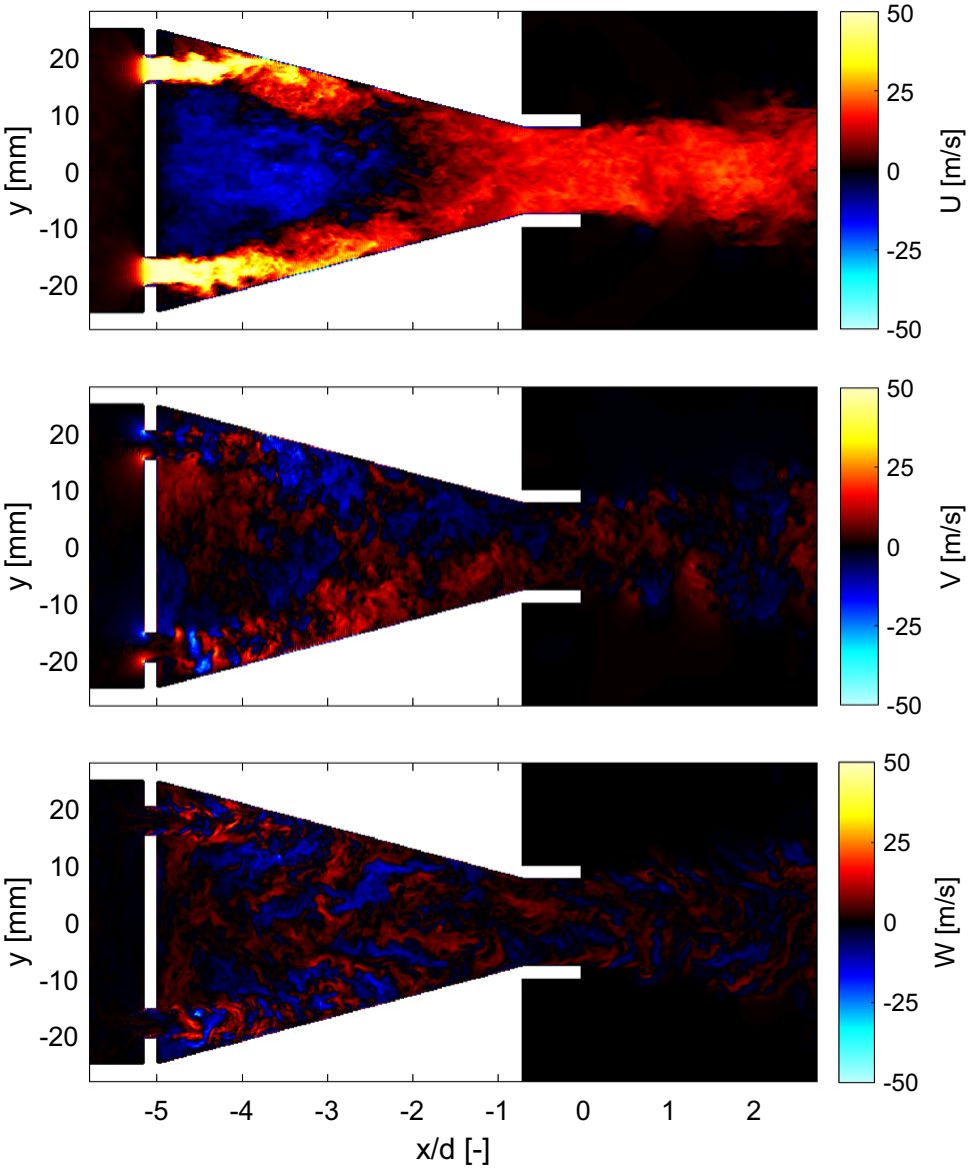
Instantaneous velocity fields at the center longitudinal plane of the burner along the axial (top), radial (middle) and azimuthal directions (bottom)

To get an insight of the turbulence generation mechanism in the burner, Fig. 9 shows instantaneous velocity fields computed with LES at the middle longitudinal plane of the burner along the axial, radial, and azimuthal directions (*U*, *V* and *W*, respectively) at atmospheric pressure. The velocity components were extracted after the initial transient startup of the simulation and the flow had reached a quasi-steady state (*t* = 385 ms). All results are spatially referred to a *xy*-coordinate system located at the main nozzle exit of the burner (see Fig. 1). From the axial component of the velocity field (Fig. 9, top), it can be seen that the air flowing from the mixing section forms jets through the openings of the turbulence generator plate which impinge the wall of the conical contraction. This results in a recirculation zone in the middle of the contraction and relatively high-velocity regions along the wall that persist almost until the exit. No signs of flow separation are observed along either the converging wall or the straight section of the nozzle. The magnitude of the instantaneous radial and azimuthal components of the velocity (Fig. 9 middle and bottom, respectively) are much lower than the one of the axial component. Strong local fluctuations of the direction of the radial and azimuthal components of the vectors are observed in the whole field which is associated with turbulent vortex structures.

**Fig. 10:**
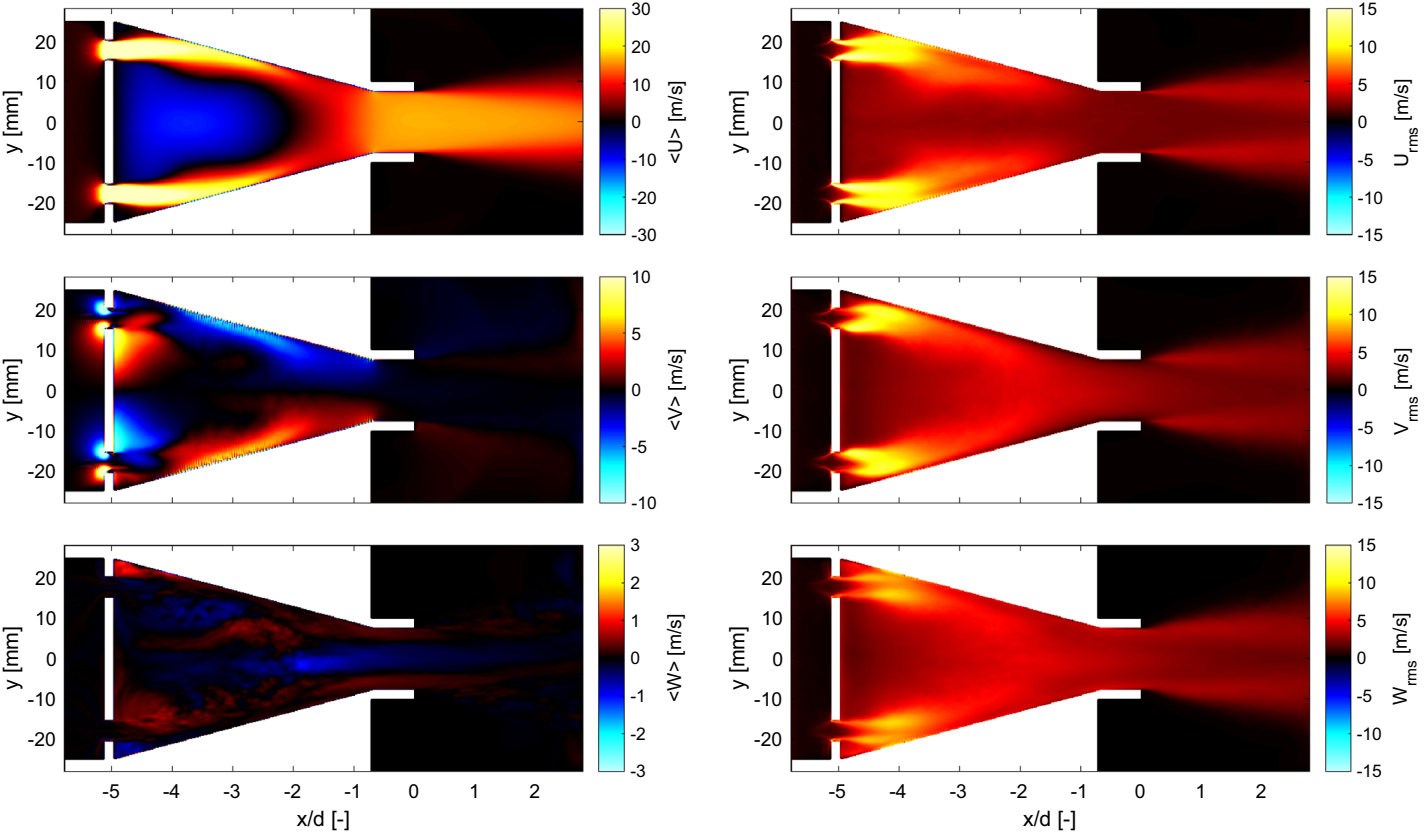
Left: Mean velocity fields along the axial (top left), radial (middle left) and azimuthal (bottom left) directions. Right: corresponding rms of the fluctuating velocity along the axial (top right), radial (middle right) and azimuthal (bottom right) directions

The observations derived from instantaneous results are consistent with the mean fields of the velocity components plotted in Fig. 10 (left). The recirculation zone formed after the turbulence generator plate extends approximately three fifths of the length of the conical contraction (Fig. 10 top left). The jets impinging the walls of the nozzle decay at the end of the conical section and a highly uniform flow is formed in the straight section of the piece. There is no sign of flow separation, and all velocity components exhibit good symmetry. By comparing the magnitude of the three components of the mean velocity ($<\!U\!>$, $<\!V\!>$ and $<\!W\!>$), it is clear that, as expected, the axial mean velocity dominates the flow at the burner exit. The root-mean-square (rms) fields of the axial, radial and azimuthal velocity components are plotted in Fig. 10 (right). The examination of the magnitude of the fluctuations in the three directions reveals a highly turbulent and nearly isotropic flow is at the burner exit.

### Turbulent flow fields at elevated pressure

The predictability of the generated turbulent flow was demonstrated in the previous section at atmospheric conditions. However, the main objective of this Bunsen burner is to generate high-quality turbulent flows at elevated pressure to mimic operating conditions of practical combustion devices. Therefore, high-spatial resolution PIV measurements were used to evaluate the turbulent flow properties at the burner exit for different high-pressure conditions and for varying bulk velocities.

**Fig. 11:**
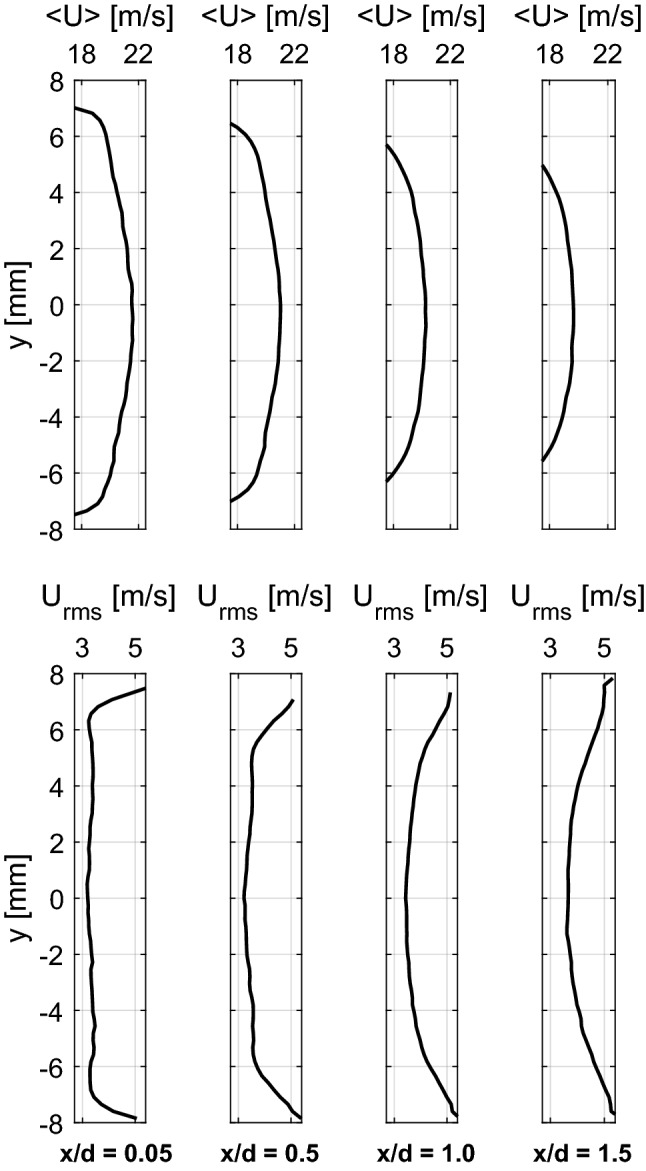
Mean (top) and rms (bottom) radial profiles of the axial velocity component at the middle longitudinal plane of the burner and at different axial locations for non-reacting flow at *P* = 5 bar and $U_0$ = 20 m/s

Figure 11 shows the mean and rms radial profiles of axial velocity, at the middle longitudinal plane of the burner and at different axial locations, for a non-reacting flow at *P* = 5 bar and $U_0$ = 20 m/s. The high symmetry of the axial velocity and its fluctuations is evident. The profile of the mean axial velocity near the burner exit is fairly uniform, representative of a mature turbulent flow. $U_{rms}$ exhibits fairly flat radial distribution within the jet core (Fig. 11 bottom) with a magnitude of $\sim$3 m/s. This means that the turbulence intensity ($U_{rms}/U_0$) around the jet center is fairly homogeneous and it lies around 15% for this operating condition.

**Fig. 12:**
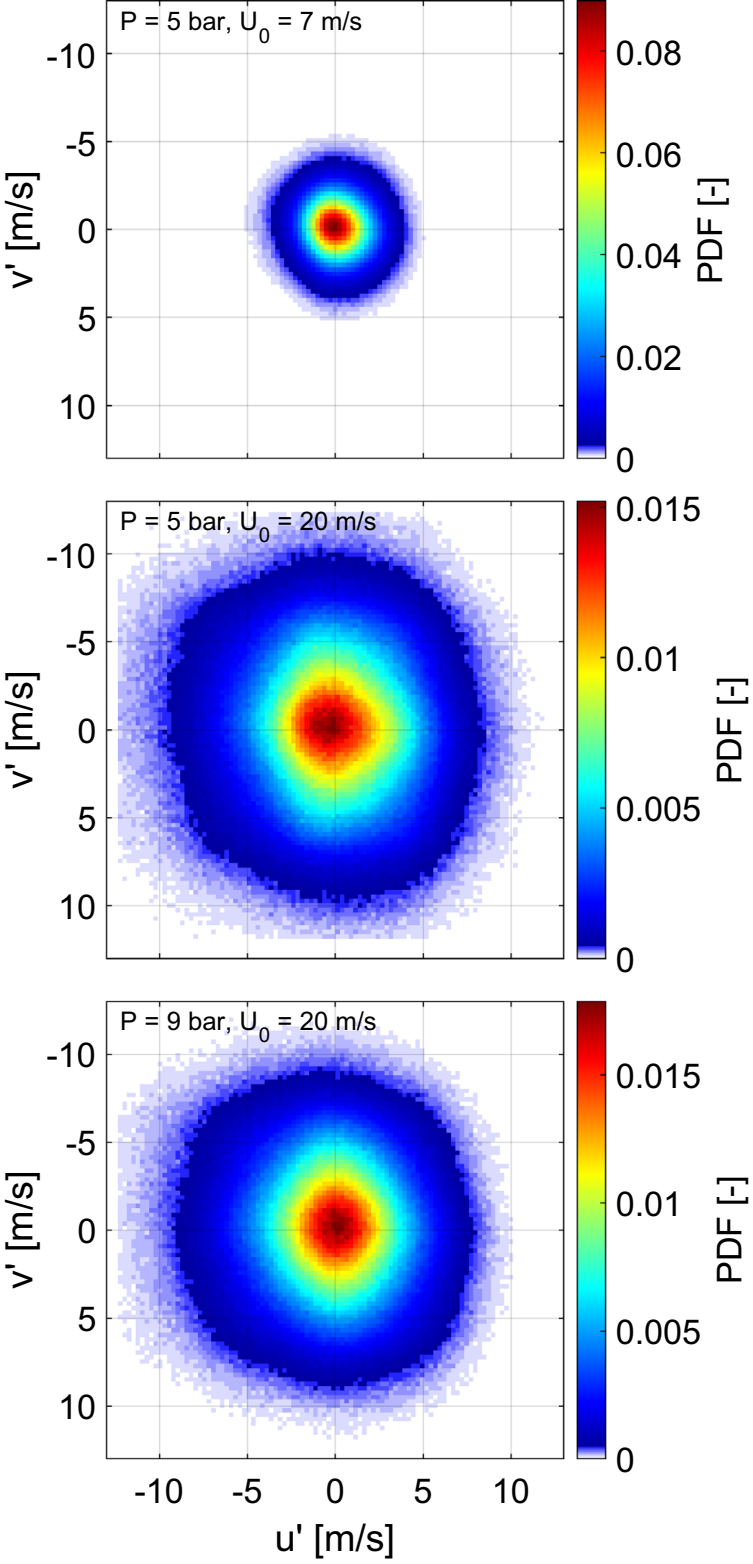
Joint PDF of $u'$ vs $v'$ at high pressure conditions: 5 bar and $U_0$ = 7 m/s (top), 5 bar and $U_0$ = 20 m/s (middle) and 9 bar and $U_0$ = 20 m/s (bottom)

Following the approach described in the previous section, the isotropy of the turbulent flows generated at elevated pressure and different bulk velocities was evaluated at the burner exit by analyzing the joint PDF of axial and radial velocity fluctuations displayed in Fig. 12. For each condition, 1000 statistically independent single shots were used for the computation. As it can be seen, at 5 bar and a bulk velocity of 7 m/s (Fig. 12 top), the flow generated at the burner exit exhibits a high degree of isotropy which was maintained when increasing the pressure and bulk velocity (Fig. 12 middle and bottom).

**Fig. 13:**
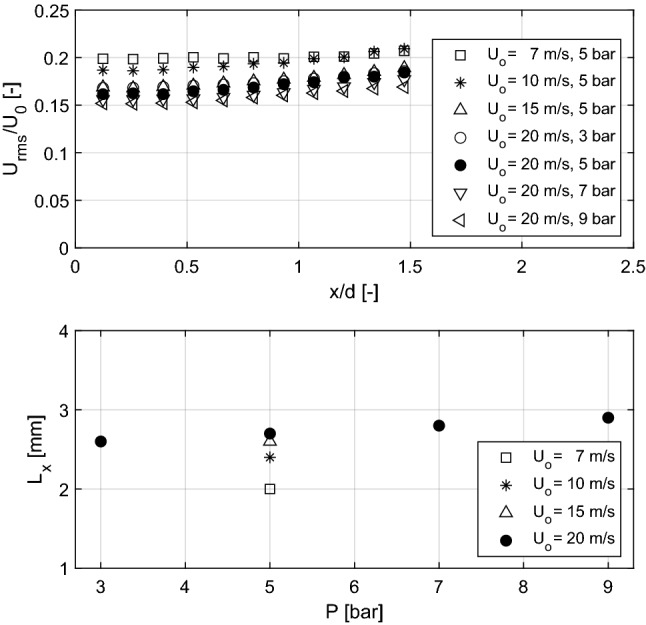
Top: turbulence intensity along the centerline of the field at different distances from the nozzle exit for different operating conditions. Bottom: longitudinal integral scale a *x*/*d* = 0.5 for different pressures and bulk velocities

Figure 13 (top) summarizes the results of the turbulence intensity calculated along the centerline of the flow field at several distances from the nozzle exit, for varied pressure conditions and bulk velocities. The turbulence intensity of the flows generated with the Bunsen burner at elevated pressure and varied exit velocities remains relatively constant within the evaluated area, ranging between 15 and 20%.

The $L_x$ integral scale was computed from the PIV results for the different operating conditions using the spatial autocorrelation function in Eq. (4), for a point on the centerline and *x*/*d* = 0.5. As can be seen in Fig. 13 (bottom), $L_x$, pressure and exit flow velocity have a negligible effect on the longitudinal integral scale for the range of operating conditions of the present study.

As discussed above, the integral length scale is one of the most important parameters to characterize turbulent flows and turbulent flames (see Eq. (1) to (3)). Therefore, the control of the properties of the turbulent flows generated with the DLR Bunsen burner (e.g., turbulence intensity and $L_x$) can enable a systematic control of the turbulent dimensionless numbers ($Re_T$, $Da_{T,P}$ and $Ka_{T,P}$) and therefore of the regime at which the premixed combustion takes place. This is essential for comprehensive studies of turbulent flames at elevated pressure. Table 2 summarizes the turbulent Reynolds number at the different elevated pressure conditions for the non-reacting flow of the present study. Flows with near-homogeneous and mature turbulence, at intermediate and extreme turbulence levels (but with similar turbulence intensities and length scales), can be obtained with the present burner at elevated pressures of interest for premixed combustion research.

**Table 2:** Resulting turbulent Reynolds number $Re_{T}$ [-] at different operating conditions

*P* [bar]	$U_0$ [m/s]			
	7	10	15	20
3	-	-	-	1780
5	940	1500	2240	3000
7	-	-	-	4100
9	-	-	-	5320

## Summary and conclusions

A new burner for the study of high-turbulence premixed flames at elevated pressures has been described and the generated turbulent flow fields have been characterized for non-reacting flows. To get an insight of turbulence generation in the burner and evaluate the predictability of the experimental data, the inner flow through the burner was computed using LES at atmospheric pressure, and the resulting mean flow field at the burner exit was compared with experimental results from high-speed PIV in terms of radial and axial profiles of the velocity components and their fluctuations. The turbulent flow fields were further experimentally characterized at elevated pressure conditions for different bulk velocities.

The data and analysis presented in this study demonstrate the burner yields a computationally reproducible approximation of homogeneous, isotropic turbulence the nozzle exit, which becomes even more isotropic with increasing downstream distance. Mean and fluctuating velocity profiles, turbulence intensities and integral scales of non-reacting flows generated with the present Bunsen burner can be well predicted with LES. At atmospheric pressure, the turbulence intensity of the flow was measured to be between $\sim$11-14% at the burner exit. A longitudinal integral scale of $\sim$2 mm was measured at the same location. The results additionally showed that the variation of pressure and bulk velocity did not have a significant effect on the turbulence intensity nor on the integral scale of the turbulent flow. Near the burner exit, those parameters ranged between 15-20 % and 2-3 mm, respectively. These findings imply that for future studies, simulations of the inner flow through the burner should not be required when varying the operating conditions of the burner. Future studies should, however, account for the confinement chamber and pilot flow downstream of the nozzle exit.

The burner has potential of allowing well-controlled systematic variation of combustion parameters and to reach intermediate and extreme turbulence levels at elevated pressure. For the conditions of the present study, turbulent Reynolds numbers up to 5300 were obtained at elevated pressure and moderate bulk velocities. We note that since the completion of the present study, flames of hydrogen-enriched natural gas have been measured using this burner at pressures of up to 10 bars, with turbulence Reynolds number of up to 10,000 and Karlovitz numbers of up to 47. The upper limit of operability is not yet known. This can enable the study of highly turbulent premixed flames at laboratory scale to generate relevant experimental data for the validation, improvement and development of combustion models at conditions similar to those of real combustion devices.
